# Environmental influences on light response parameters of net carbon exchange in two rotation croplands on the North China Plain

**DOI:** 10.1038/s41598-019-55340-2

**Published:** 2019-12-10

**Authors:** Xueyan Bao, Zhigang Li, Futi Xie

**Affiliations:** 1 Agricultural Collage, Inner Mongolia University for Nationalities, Tongliao, China; 2 Agricultural Collage, Shenyang Agricultural University, Shenyang, China

**Keywords:** Ecosystem ecology, Climate-change ecology

## Abstract

The ecosystem light response parameters, i.e. apparent quantum yield (*α*), maximum rate of ecosystem gross photosynthesis (*A*_*max*_), and daytime ecosystem respiration (*R*_*d*_), are very important when estimating regional carbon budgets. But they are not well understood in double cropping systems. Here, continuous flux data were collected from two rotation croplands in Yucheng (YC) and in Luancheng (LC) to describe the among-year variations in *α*, *A*_*max*_, and *R*_*d*_, and to investigate variation mechanism on an annual scale. The three parameters exhibited marked fluctuations during the observation years. The annual *α*, *A*_*max*_, and *R*_*d*_ ranged from 0.0022–0.0059 mg CO_2_ μmol photon^−1^, from 2.33–4.43 mg CO_2_ m^−2^ s^−1^, and from 0.19–0.47 mg CO_2_ m^−2^ s^−1^ at YC, and from 0.0016–0.0021 mg CO_2_ μmol photon^−1^, from 3.00–6.30 mg CO_2_ m^−2^ s^−1^, and from 0.06–0.19 mg CO_2_ m^−2^ s^−1^ at LC, respectively. Annual *α* and *R*_*d*_ declined significantly when vapor pressure deficit (VPD) exceeded 1.05 kPa and increased significantly when canopy conductance (*g*_*c*_) exceed 6.33 mm/s at YC, but changed slightly when VPD and *g*_*c*_ exceeded 1.16 kPa and 7.77 mm/s at LC, respectively. The fact that the negative effects of VPD and *g*_*c*_ on *α* and *R*_*d*_ at LC were not as significant as they were at YC may be attributed to different climate conditions and planting species. A negative relationship (R^2^ = 0.90 for YC and 0.89 for LC) existed between VPD and *g*_*c*_. Therefore, the VPD, through its negative effect on *g*_*c*_, inhibited *α* and *R*_*d*_ indirectly. Among-year *A*_*max*_ variation was mainly influenced by the annual mean surface soil temperature (T_s_) of non-growing season of wheat significantly (R^2^ = 0.59, P < 0.01). Therefore, in future climate change scenarios, these environmental effects need to be included in carbon cycle models so that the accuracy of the carbon budget estimation can be improved.

## Introduction

Recently, a large number of studies began to focus on the carbon (CO_2_) exchange flux at ecosystem level with the eddy covariance (EC) technique^1–4^. The net ecosystem carbon exchange (NEE) obtained from the eddy towers often shows high dependence on the photosynthetic proton flux density (PPFD), which can be described well by the Michaelis-Menten rectangular hyperbola model^5^. This model is extremely important because it is often utilized in gap filling strategies when there is missing flux data^6,7^. Moreover, deriving this relationship is among the very first steps taken when attempting to understand the effects of potential regulating mechanisms in ecosystem studies^8^.

The Michaelis-Menten rectangular hyperbola model has three parameters: the ecosystem apparent quantum yield (*α*), maximum rate of ecosystem gross photosynthesis (*A*_*max*_), and the bulk daytime ecosystem respiration at PPFD = 0 (*R*_*d*_). *α* is the maximum use of PPFD and the initial slope of the NEE-PPFD rectangular curve^6^. It reflects the utilization efficiency of weak light for ecosystems and biochemical characteristics of photosynthesis^9^. *A*_*max*_, the value of asymptote of the light response curve, represents the light – saturated rate of CO_2_ assimilation^10^ and is an indicator of activity of the photosynthetic system in plants^10,11^. Approximately 50% of the photosynthate is back to atmosphere through canopy dark respiration (*R*_*d*_)^12^, so R_d_ is an essential part of ecosystem carbon cycling. Because of their important roles in affecting the shape of NEE light response curves and attempting to examine the balance between plant photosynthesis and respiration^13^, the three parameters have been extensively researched as part of the overall assessment of the global carbon budget^14^.

Many studies have discussed the variations in ecosystem *α, A*_*max*_, and *R*_*d*_^4,15^, and their influencing factors^16,17^. The ecosystem *α*, A_max_, and R_d_ could be affected by vapor pressure deficit (VPD)^3,17–19^, soil water content (SWC)^1,20,21^, temperature^4,22^, and biotic factors, such as leaf area index (LAI) and canopy conductance (*g*_*c*_)^15^. The variations in ecosystem *α*, *A*_*max*_, and *R*_*d*_ usually show similar patterns and coincide with the trends of canopy development on a seasonal scale^18^. Some studies indicated that the light response parameters were stimulated under elevated air temperature (T_a_)^4,18,23^, but other studies showed no relationship between them probably due to the photosynthate allocation and stomatal factors^3^. Dry condition, which is commonly characterized by high temperature, high VPD and deficient surface soil water, can greatly inhibit the parameters by promoting stomatal closure^21^, but some ecosystems were not affected by high VPD due to their tolerance of drought stress^20^.

Previous studies provided insight into the characteristics of NEE-PPFD relationship in terrestrial ecosystems under the scenario of global change. However, most studies were conducted over a single growing season per year in forests^18,24^, grassland^3,4^, and cropland^21^, which means that the fluctuation patterns for the parameters and the affecting mechanism in double cropping systems are still unknown. However, it is necessary to explore the light response characteristics of carbon exchange in such ecosystems because field management processes, such as frequent tilling and residue return, in a double cropping system could directly affect the soil organic carbon content and soil microorganism biomass^25,26^, both of which can influence the carbon cycle in double cropping agricultural areas. Furthermore, most previous studies that focused on agreoecosystem were based on short term flux databases (1~3 years). For example, Stirling, *et al*.^27^ investigated changes in the light response curve and parameters only during leaf development stage of maize. Zhang, *et al*.^20^ examined the effect of environmental factors on the light response parameters based on one year flux data. Zhang, *et al*.^3^ used three consecutive years to evaluate the seasonal variations in the light model parameters and the underlying mechanism. The short – term flux measurement can not produce statistical and reliable relationship between the parameters and potential affecting factors, so more long-term flux data are needed to improve our understanding of how the carbon exchange rate response to a wide range of different factors^28^.

In China, croplands cover the third largest area after forests and grassland^29^. The agroecosystem is easier to be affected by human activities than other ecosystems because farm land is intensively managed by humans^30^ who can increase carbon uptake through efficient crop management practices^31^. Therefore, agriculture is considered to be a strong contributor to the regional carbon budget^32^. The North China Plain is 3 × 10^5^ km^2^ in size^33^. It is the largest agricultural production center in China and occupies approximately 18.6% of the total national agricultural area^21^. The North China Plain provides more than half of the wheat and about 33% of maize consumed in China^34^. A winter wheat-summer maize rotation cropping system is the main planting pattern in this area. Its large area and its importance to national economics mean that the North China Plain has a considerable effect on the regional carbon balance^35,36^. Therefore, exploring the photosynthetic features of the agricultural ecosystem in this region will help improve carbon cycle models and the accuracy of future net ecosystem carbon exchange predictions. Based on CO_2_ flux and micrometeorological measurements, the objectives of this study were to (1) describe the among-year variations in annual ecosystem *α, A*_*max*_, and *R*_*d*_ by using continuous eddy covariance data for Yucheng (YC) from 2003 to 2012 and Luancheng (LC) from 2008 to 2012; and (2) analyze how environmental and biotic factors affect the annual ecosystem *α*, *A*_*max*_, and *R*_*d*_.

## Results and Discussion

### Variations in environmental and biotic factors

**Figure 1:**
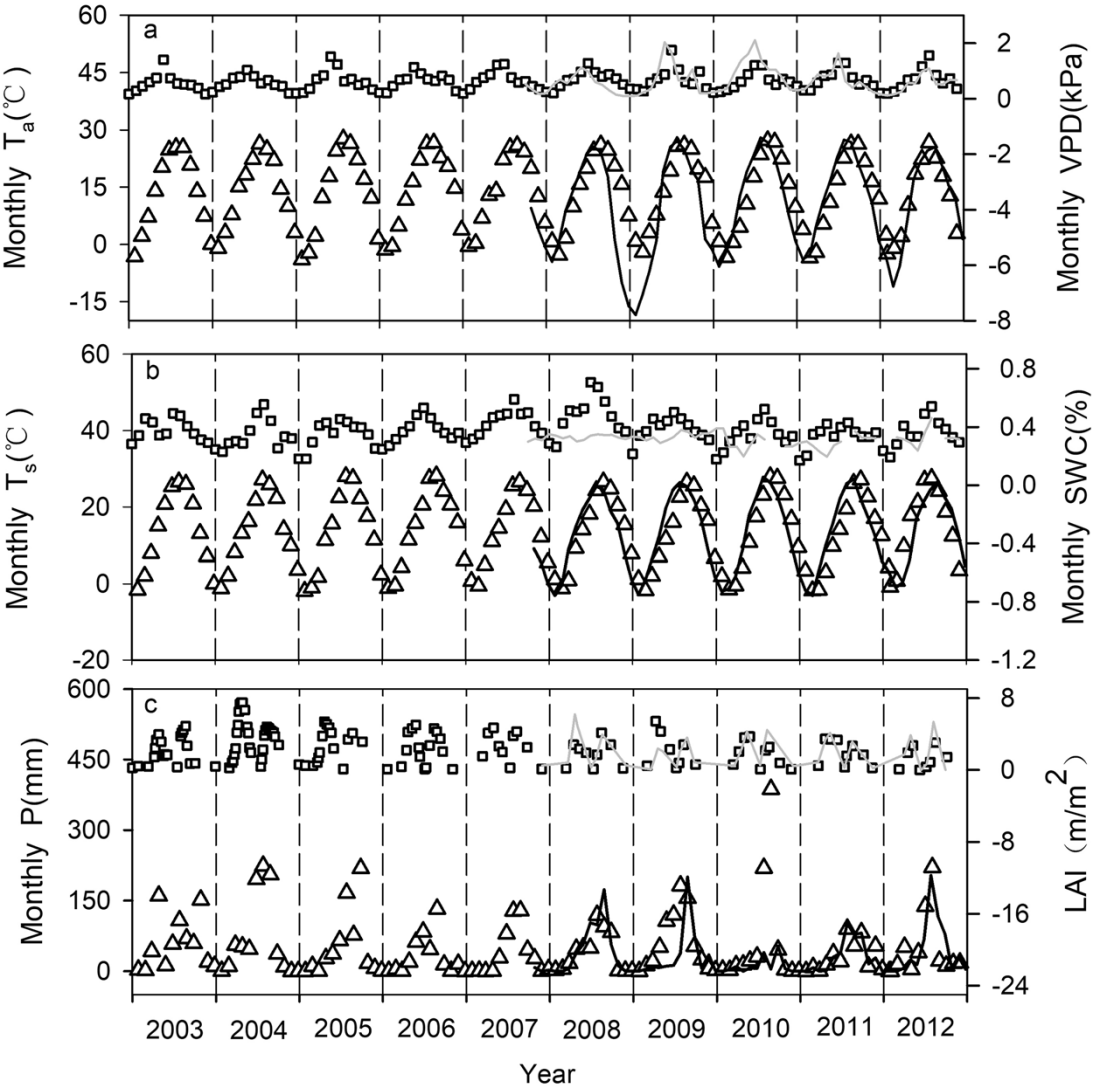
Seasonal and interannual variations in meteorological and phenological variables. The triangles and black solid lines in Figures (**a**,**b** and **c**) represent the monthly mean air temperature (Monthly T_a_, °C), monthly mean soil temperature (Monthly T_s_, °C) at 5 cm depth, and the monthly accumulated precipitation (P, mm) at the YC and LC sites, respectively. The squares and gray solid lines in Figures (**a**,**b** and **c**) represent the monthly mean vapor pressure deficit (Monthly VPD, kPa), monthly mean soil water content (Monthly SWC, %), and the leaf area index (LAI, mm^−2^) at the YC and LC sites, respectively

Figure 1 shows the seasonal and interannual patterns for monthly environmental factors, and the LAI at the YC and LC sites. The maximum and minimum values for the air and soil temperatures (T_s_) occurred between July and August and between November and January of the next year, respectively. The annual mean air temperature (M-T_a_) varied from 11.5 °C to 13.9 °C with a multi-year mean of 12.9 ± 0.7 °C (mean ± standard deviation) at the YC site and from 8.0 °C to 12.7 °C with a multi-year mean of 10.4 ± 2.2 °C at the LC site during the observation years. The annual mean for T_s_ varied from 11.2 °C to 13.5 °C at the YC site and from 12.5 °C to 13.6 °C at the LC site. The straw residue from the summer maize remained in the field after harvesting. Therefore, the T_s_ decrease was not as great as the T_a_ decrease when extreme cold weather occurred (Fig. 1a,b). As a result, the T_s_ was higher than the T_a_ in 2008–2009 and 2011–2012 at the LC site. Generally, the seasonal pattern for the monthly mean VPD had a single-peak curve at both sites, except for 2009 at the YC site. The annual mean VPD varied from 0.57 to 1.14 kPa at the YC site and from 0.32 to 1.07 kPa at the LC site during the observation years (Fig. 1a). Precipitation mainly occurred from June to September every year and the annual accumulated value was 528.3 mm and 399.5 mm at the YC and LC sites, respectively (Fig. 1c). The seasonal SWC variation often produced two peaks in one year, which occurred during the two growing seasons, respectively. The peak summer maize SWC value was higher than that for winter wheat, partially because of the routine irrigation during the growing season and the high precipitation frequency in summer. The annual mean SWC ranged from 0.34 to 0.48 m^3^ m^−3^ and from 0.29 to 0.33 m^3^ m^−3^ during the observation years at the YC and LC sites, respectively (Fig. 1b). The LAI was relatively low during the period from late October to mid-March of the next year, but then gradually increased to reach a maximum in May. The maximum LAI value for summer maize often occurred in August (Fig. 1c). Therefore, the relevant meteorological and biotical factors showed marked interannual variability, providing an opportunity to study the underlying mechanisms of the interannual variations in light response parameters, which will be discussed later in this paper.

### The light response characteristic of NEE and a comparison with other studies

**Figure 2:**
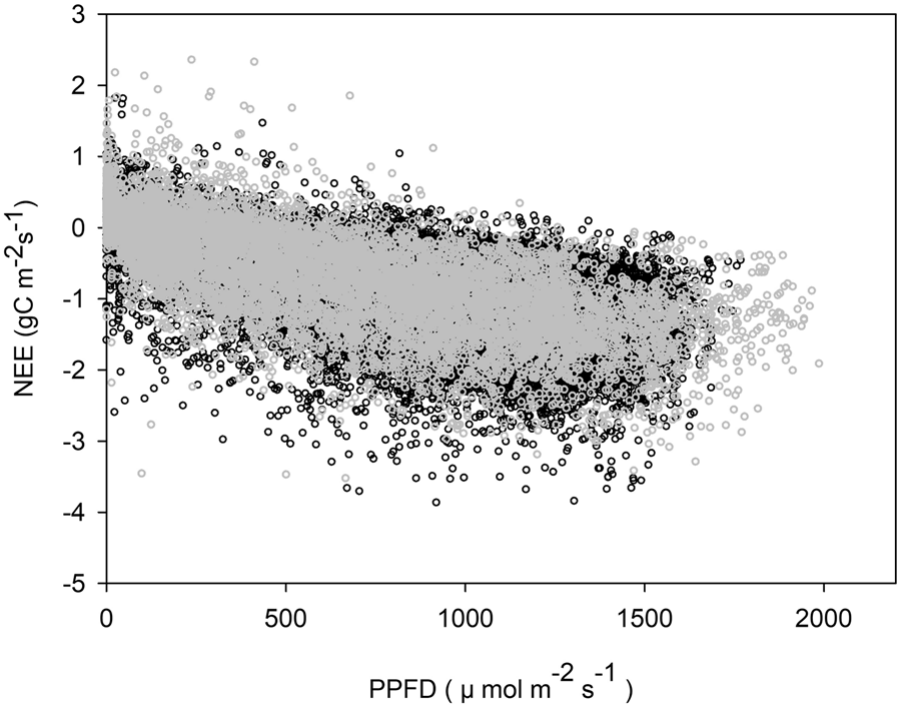
Net ecosystem carbon exchange (NEE) (g C m^−2^ s^−1^) responses to PPFD (μ mol m^−2^ s^−1^) at a half-hour scale. Black dots denote incident NEE at YC from 2003–2012 and gray dots denote incident NEE at LC from 2008–2012.

**Figure 3:**
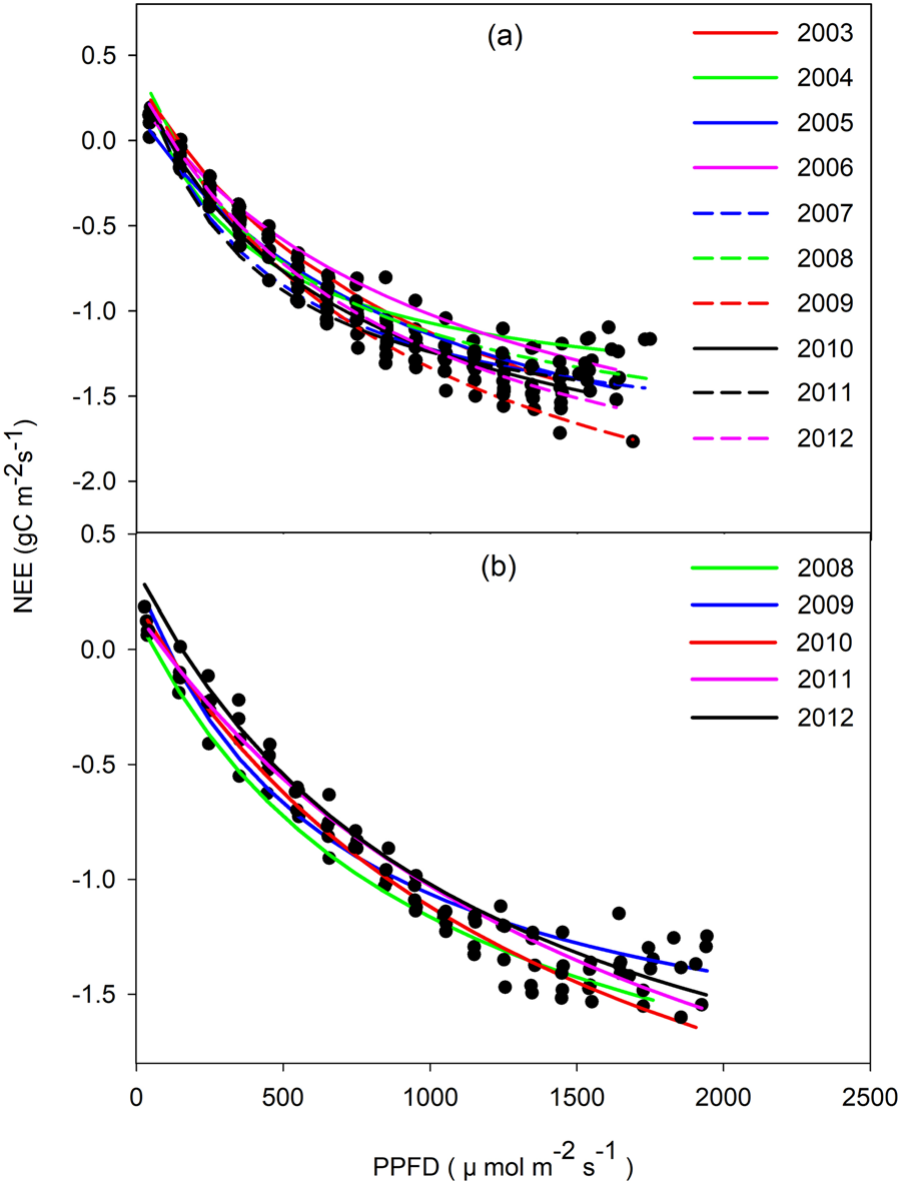
Light response curves fitted by the collective average for incident valid daytime PPFD (μ mol m^−2^ s^−1^) and NEE (gC m^−2^ s^−1^) at a half-hour scale for YC (**a**) and LC (**b**). The average value was calculated within a 100PPFD interval.

The parameters of the light response model exhibited distinct variations among years. Figures 2 and 3 show the relationship of NEE-PPFD at a half hourly scale from 2003–2012 and from 2008–2012 at the YC and LC sites, respectively. The absolute value of NEE (│NEE│, namely the carbon absorbed by the ecosystem) rose as the PPFD increased at low-to-intermediate PPFD levels, but │NEE│began to decline when the PPFD exceeded the light saturation point. Table 1 lists the annual ecosystem light response parameters derived from Eq. 2 using the collective averages for incident valid daytime PPFD and NEE. The annual ecosystem *α*, *A*_*max*_, and *R*_*d*_ ranged from 0.0022–0.0059 mgCO_2_ μmol photon^−1^, from 2.33–4.43 mg CO_2_ m^−2^ s^−1^, and from 0.19–0.47 mg CO_2_ m^−2^ s^−1^ between 2003 and 2012 at the YC site, respectively. The annual ecosystem *α*, *A*_*max*_, and *R*_*d*_ at the LC site ranged from 0.0016–0.0021 mg CO_2_ μmol photon^−1^, from 3.00–6.30 mg CO_2_ m^−2^ s^−1^, and from 0.06–0.19 mg CO_2_ m^−2^ s^−1^ between 2008 and 2012, respectively (Table 1). The multi-year means for annual ecosystem *α* and *R*_*d*_ were 0.0032 ± 0.0011 mg CO_2_ μmol photon^−1^ and 0.31 ± 0.077 mg CO_2_ m^−2^ s^−1^ at the YC site, respectively, which were higher than those recorded at the LC site (0.0018 ± 0.00018 mg CO_2_ μmol photon^−1^ for *α* and 0.13 ± 0.056 mg CO_2_ m^−2^ s^−1^ for *R*_*d*_). However, the long term mean for annual ecosystem *A*_*max*_ at the YC site (3.15 ± 0.62 mg CO_2_ m^−2^ s^−1^) was lower than at the LC site (4.40 ± 1.22 mg CO_2_ m^−2^ s^−1^). The annual ecosystem *α* for the YC site was slightly higher than the results obtained for a winter wheat field in central Germany^32^ and a spring wheat field in Canada^37^ (0.0028 mg CO_2_ μmol photon^−1^ and 0.0016 mg CO_2_ μmol photon^−1^, respectively), but were still within the moderate range. The annual ecosystem *A*_*max*_ for the YC and LC sites in this study were also higher than the results obtained for a winter wheat field in central Germany^32^ and a spring wheat field in Canada^37^ (2.78 mg CO_2_ μmol photon^−1^, 3.01 mg CO_2_ μmol photon^−1^, respectively). However, the combined effects of the differences in raw data processing, curve fitting methods, environmental conditions, species composition, the photosynthesis pathway in different species, and land management could explain the dissimilarities in light response characteristics between different ecosystems or regions^38^.

**Table 1:** Annual values for the light response parameters: *α* (mg CO_2_ μmol photon^−1^), *A*_*max*_ (mg CO_2_ m^−2^ s^−1^), and *R*_*d*_ (mg CO_2_ m^−2^ s^−1^) when estimated by the Michaelis-Menten rectangular hyperbola equation.

Sites	Year	*α* (mg CO_2_ μmol photon^−1^)	*A*_*max*_ (mg CO_2_ m^−2^ s^−1^)	*R*_*d*_ (mg CO_2_ m^−2^ s^−1^)	R^2^
YC	2003	0.0029	3.06	0.37	0.93**
	2004	0.0030	2.87	0.28	0.99**
	2005	0.0022	3.14	0.19	0.93**
	2006	0.0023	2.79	0.24	0.98**
	2007	0.0039	2.76	0.33	0.97**
	2008	0.0023	4.43	0.29	0.83**
	2009	0.0031	3.85	0.28	0.98**
	2010	0.0037	2.78	0.35	0.98**
	2011	0.0059	2.33	0.47	0.99**
	2012	0.0028	3.46	0.29	0.98**
	Mean	0.0032	3.15	0.31	
	Std	0.0011	0.62	0.077	
LC	2008	0.0018	3.84	0.06	0.91**
	2009	0.0019	4.30	0.17	0.96**
	2010	0.0016	6.30	0.09	0.86**
	2011	0.0021	3.00	0.19	0.95**
	2012	0.0018	4.58	0.16	0.96**
	Mean	0.0018	4.40	0.13	
	Std	0.00018	1.22	0.056	

Note: Std indicates standard deviation, **indicates a significant correlation at the level of 0.01.

### Factors influencing the annual ecosystem light response parameters

#### Water conditions

**Figure 4:**
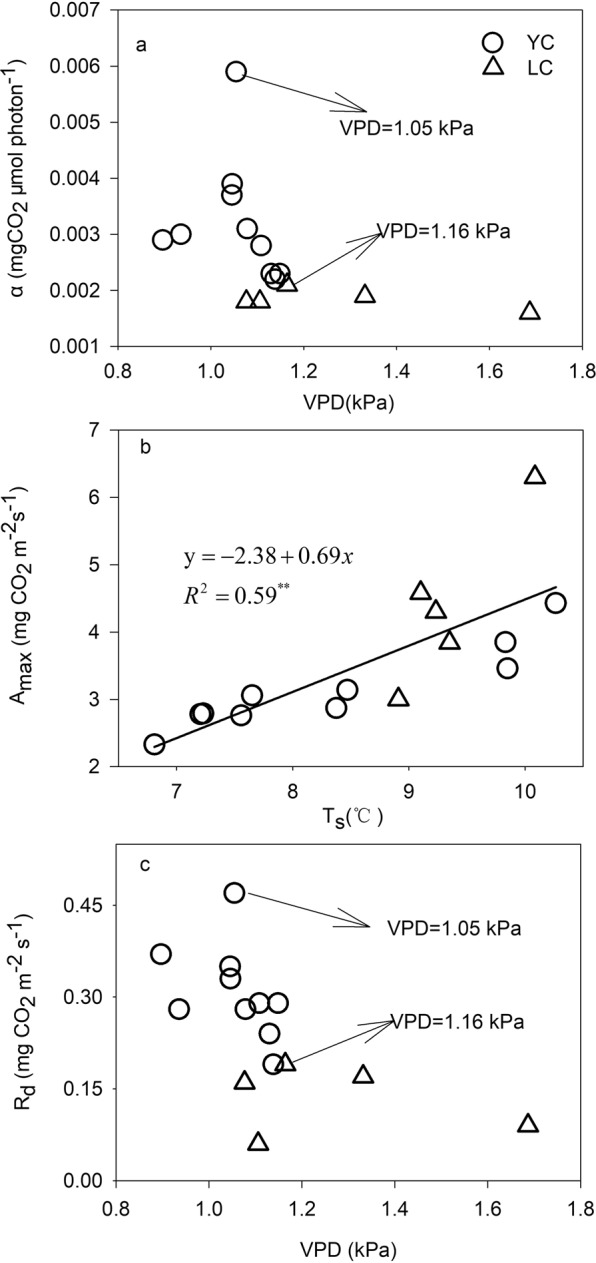
Variation trends for the annual *α* and *R*_*d*_ (**a** and **c**) relationship with the annual mean of main growing season VPD, and the annual *A*_*max*_ relationship with the annual mean soil temperature (T_s_) during the non-main growing season for winter wheat (**b**).

**Figure 5:**
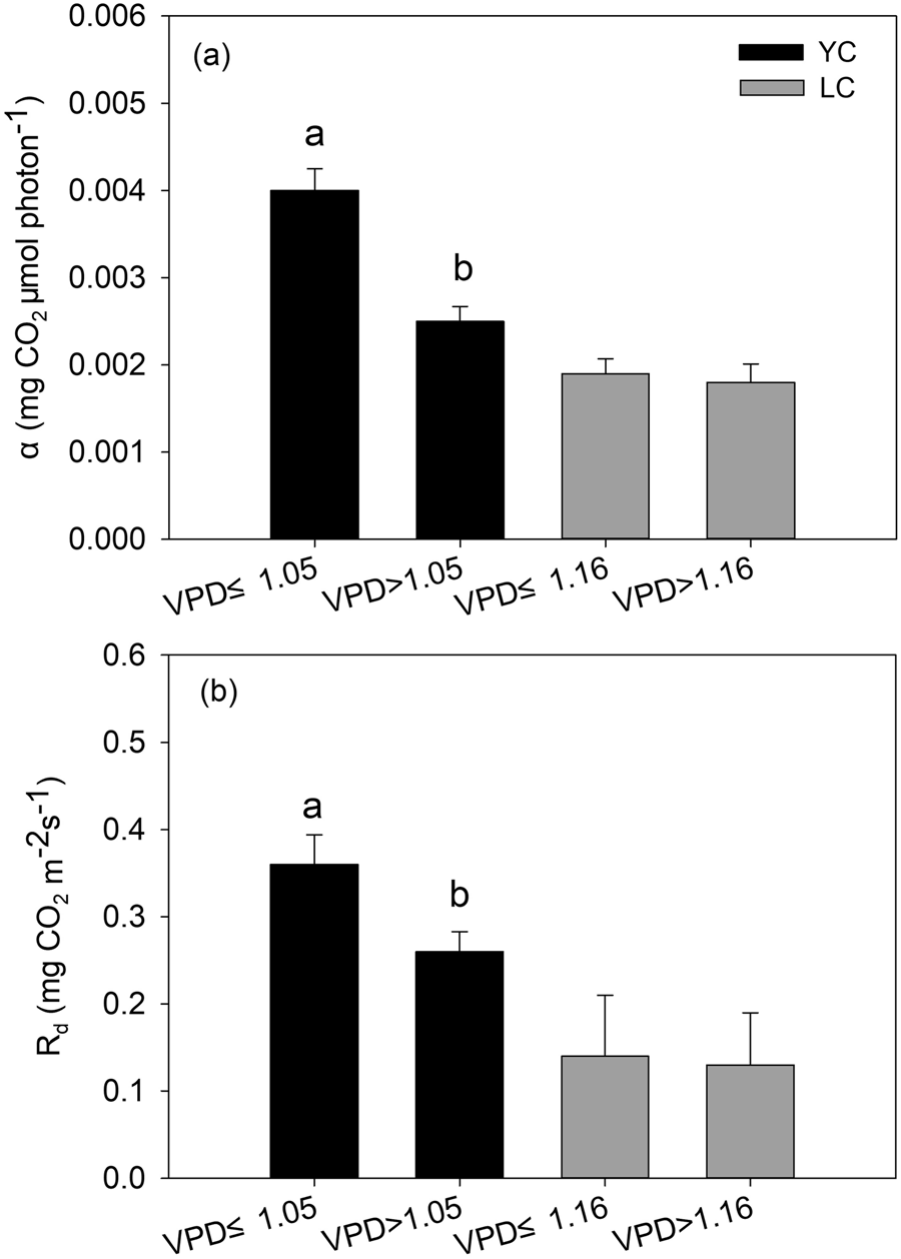
Effect of the annual mean for the main growing season VPD in annual *α* and *R*_*d*_. (**a** and **b**) above the column diagram represent the significant of difference in light response parameters under different VPD conditions at 0.05 level.

**Figure 6:**
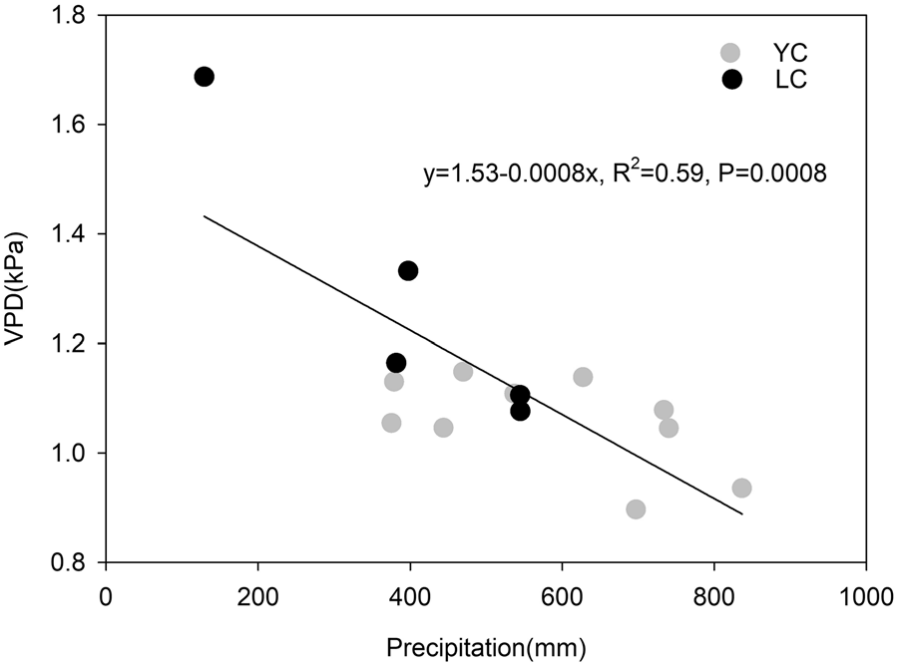
Effect of the annual precipitation on the annual mean for main growing season VPD.

Annual ecosystem *α* and R_d_ were inhibited when the annual mean for main growing season VPD was high at the YC site (Figs. 4 and 5). In this study, high VPDs commonly occur from May to July due to the low rainfall during this period, because a significant and positive relationship between annual mean for the main growing season VPD and annual accumulated precipitation was found, R^2^ = 0.59, P = 0.0008 (Fig. 6). Figure 4a shows that the annual mean VPD ranged from 0.90~1.14 kPa and from 1.08~1.69 kPa at the YC and LC sites, respectively. The maximum value for annual ecosystem *α* and *R*_*d*_ for the YC and LC sites occurred when the annual mean of main growing season VPD was 1.05 kPa and 1.16 kPa, respectively. However, the annual ecosystem *α* and *R*_*d*_ declined when the VPD exceeded the thresholds at the two sites. At the YC site, the multi-year means for annual ecosystem *α* and *R*_*d*_ when the VPD >1.05 kPa were significantly lower than when the VPD ≤1.05 kPa (P = 0.044) (Fig. 5), which indicated that VPD had negative effects on annual ecosystem *α* and *R*_*d*_. The conclusion that a high VPD limited ecosystem light response parameters was consistent with previous studies. For example, Zhang, *et al*.^18^ thought that the ecosystem *α* of a subtropical *Pinus* plantation declined significantly when high VPD happened. The results of Zhang, *et al*.^3^ showed that the NEE light response coefficients were prohibited under dry conditions accompanied by high VPD in a steppe ecosystem. A study conducted in a grassland and cropland indicated that the *α* of both ecosystem decreased with increasing in VPD^20^.

**Figure 7:**
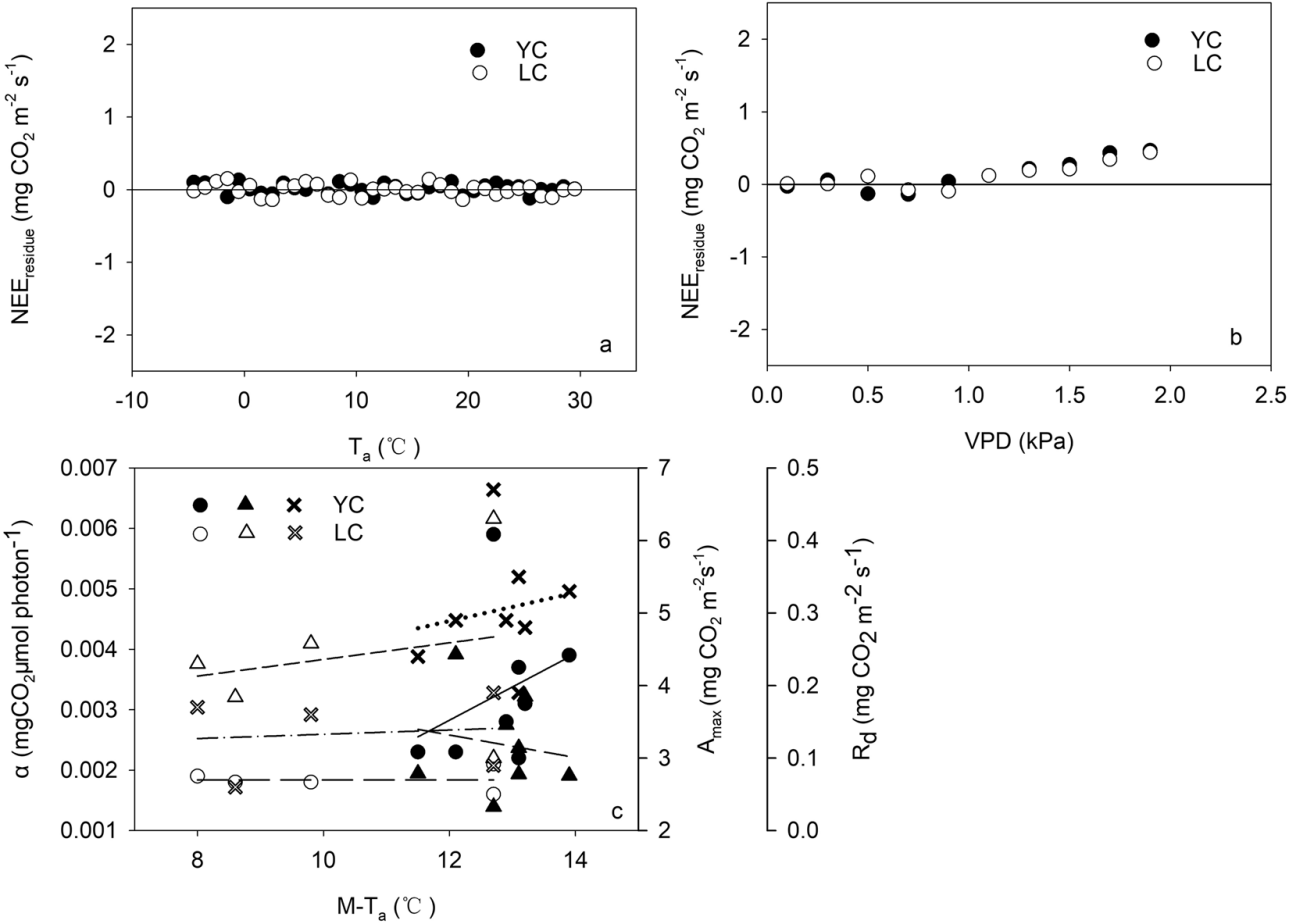
(**a**) Residuals of the data and the regression curve in Fig. 2 (NEE _residual_) vs. T_a_, the dots indicate mean for each 1 °C. (**b**) NEE _residual_ vs. VPD, the dots indicates mean for each 0.2 kPa. (**c**) Relationship between annual mean air temperature (M-T_a_) and the annual ecosystem *α, A*_*max*_ and *R*_*d*_.

There often exist high autocorrelation between T_a_ and VPD, so it is necessary to deconvolute whether the decrease in *α* and *R*_*d*_ was due to high atmosphere evaporative demand or to heat stress. This study adopted an approach that recommended by Zhang, *et al*.^18^ to distinguish which factor was more important. Figure 7a,b show the relationship between the residuals of the NEE – PPFD regression and T_a_ and VPD. The residuals did not change when T_a_ increased, but depended on VPD above 1.0–1.1 kPa. Moreover, we did not found relationship between M-T_a_ and annual light response parameters (Fig. 7c). The reason that no change of residuals with T_a_ may be related to that the drought stress was not prone to occur under the heat conditions at the two sites. In a word, the relationship between the residuals and VPD indicated VPD played an important role in depressing annual *α* and *R*_*d*_ compared with T_a_.

**Figure 8:**
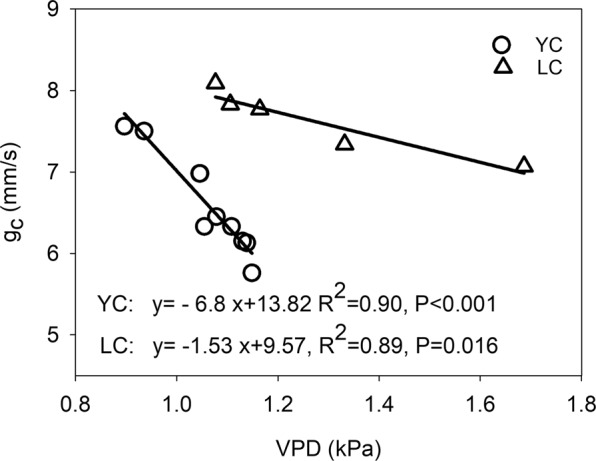
Relationships between annual means for main growing season VPD (kPa) and canopy conductance (*g*_*c*_, mm/s) at the YC and LC site.

**Figure 9:**
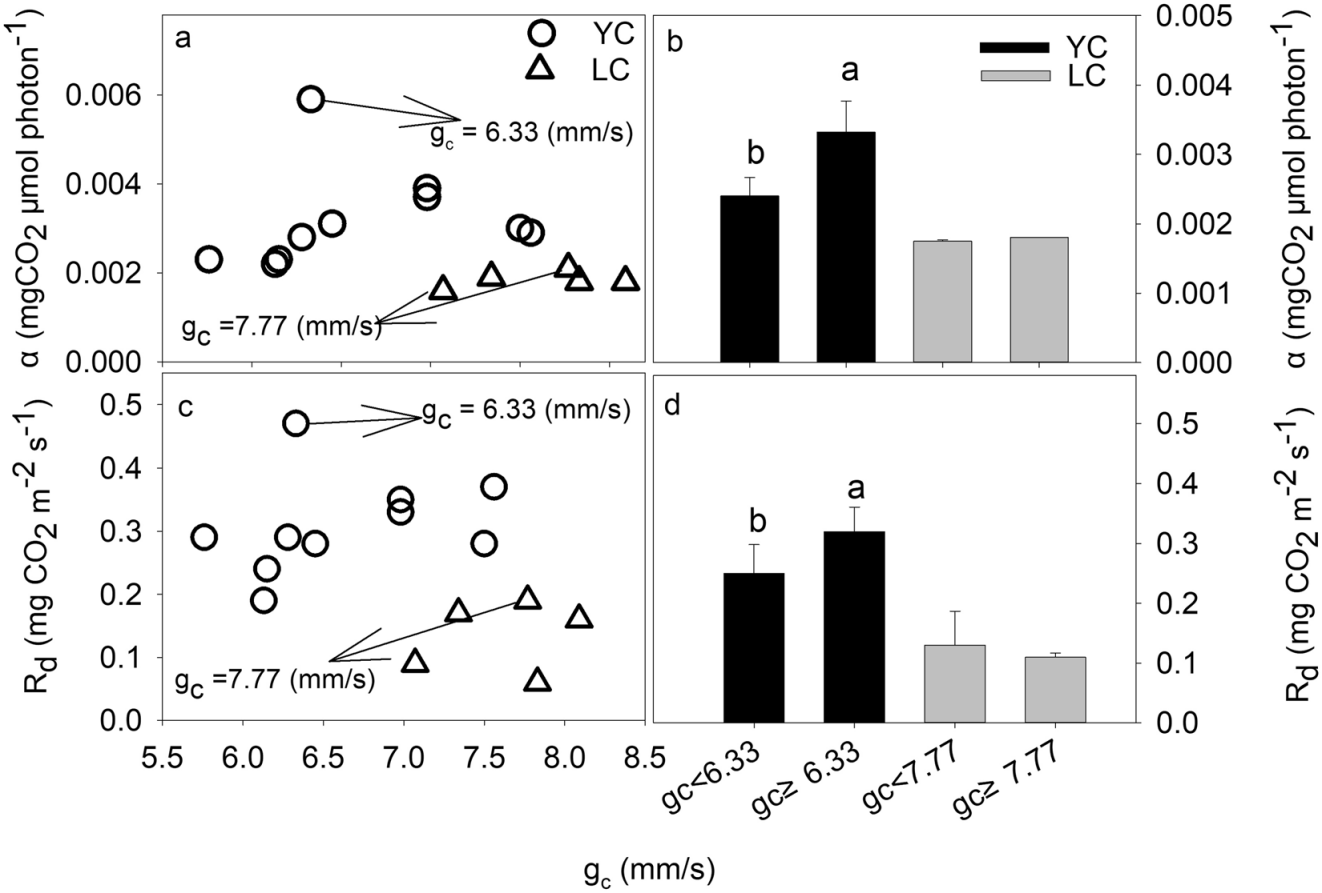
Effect of the annual mean for the main growing season canopy conductance (*g*_*c*_, mm/s) on annual *α* and *R*_*d*_. (**a** and **b**) above the column diagram represent the significant of difference in light response parameters under different *g*_*c*_ conditions at 0.05 level.

The index of *g*_*c*_ can explain why annual mean value of main growing season VPD depressed annual *α* and *R*_*d*_. It is widely accepted that a high VPD would strongly limit plant photosynthesis due to stomata closure on a leaf scale^39^. Stomata closure can cause a decrease in the leaf internal CO_2_ concentration, which leads to a decline in net CO_2_ uptake and an imbalance between photochemical activity and the electron requirements for photosynthesis^40^. Stomata conductance is a parameter quantifying leaf stomata behavior, but on ecosystem scale, the stomata behavior of the whole ecosystem commonly quantified by *g*_*c*_^41^. The EC technique provides a good change to obtain the Bowen ratio (the ratio of the sensible heat flux to the latent heat flux), which is a key parameter for estimating *g*_*c*_. We found that there was a negative relationship between annual means for main growing season *g*_*c*_ and VPD (Fig. 8), which meant that a high VPD restrain the *g*_*c*_ significantly. The negative relationship between g_c_ and VPD determined that the response trends of annual *α* and R_d_ to *g*_*c*_ were similar to their responses to VPD (Fig. 9a,c). Figure 8b,d indicated that the multi-year means for annual ecosystem *α* and *R*_*d*_ when the *g*_*c*_ < 6.33 mm/s were significantly lower than the *g*_*c*_ ≥ 6.33 mm/s (P < 0.05). Therefore, the low annual ecosystem *α* and *R*_*d*_ in this study was partially result from weak annual mean *g*_*c*_, which was commonly caused by high annual mean VPD. As a result, the annual means for VPD, through its negative effect on *g*_*c*_, inhibited annual *α* and *R*_*d*_ indirectly at the YC site.

The effects of annual mean VPD and *g*_*c*_ on *α* and *R*_*d*_ at LC site were not as significant as they were at YC site. The multi-year means for annual ecosystem *α* (0.0018 mg CO_2_ μmol photon^−1^) and *R*_*d*_ (0.13 mg CO_2_ m^−2^ s^−1^) when the VPD > 1.16 kPa were slightly lower than the multi-year means for annual ecosystem *α* (0.0019 mg CO_2_ μmol photon^−1^) and *R*_*d*_ (0.14 mg CO_2_ m^−2^ s^−1^) when the VPD ≤ 1.16 kPa (Fig. 5). The *α* and *R*_*d*_ when *g*_*c*_ < 7.77 mm s^−1^ did not significantly differ from that when *g*_*c*_ ≥ 7.77 mm s^−1^ (Fig. 9c,d). The different impact of VPD or g_c_ on *α* and *R*_*d*_ between the two sites may be attributed to the climate condition and planting species. The annual rainfall at the LC site was 399.5 ± 170.0 mm (mean ± standard deviation) during 2008–2012, whereas it was 528.3 ± 197.2 mm at the YC site during 2003–2012, which meant that the climate at the LC site was more drier than the YC site, indicating that the wheat and maize cultivars planting in the LC site were the drought – tolerance cultivars. Previous studies indicated that the drought – tolerance cultivars of winter wheat have higher net photosynthetic rate under dry conditions compared with common cultivar^42,43^, this is because the decrement of photosynthesis for the drought – tolerance cultivars was smaller than common cultivars when water stress happened^43^. However, less rainfall would lead to an increase in VPD in this study, thereafter, lead to a decrease in *g*_*c*_ (Figs. 8 and 9), indicating that the photosynthetic rate of these cultivars was still higher when the *g*_*c*_ was lower. The conclusion that the VPD and *g*_*c*_ had minor effects on *α* and *R*_*d*_ at the LC site indicated that field management practice was an potential factor influencing the characteristics of ecosystem light response.

#### T_s_

Warm soil condition can not only enhance soil respiration^44^, but also impact the photosynthesis by affecting nutrient availability^45^. In this study, a significant and positive relationship was found between annual mean T_s_ for the non-main winter wheat growing season (the period from October to mid-March of the next year) and annual ecosystem *A*_*max*_ (R^2^ = 0.59, P < 0.01) (Fig. 4b), which showed that there was a lag response by the parameter to changing environmental conditions^45^. This result may be related to the long non-main growing season for winter wheat, during which the growth of the crop almost stopped. This period included the following stages: seedling growth, tillering, overwintering, green return, and standing. During this period, winter wheat LAI only changed slightly and ranged from 0.19 ± 0.015–0.21 ± 0.012 from the tillering stage to the standing stage at the YC site and from 0.17 ± 0.009–0.18 ± 0.008 at the LC site. The small change in LAI meant that the non-main growing season can be considered as the no-growth season for winter wheat. This finding was consistent with the study by Li, *et al*.^4^, who found that T_s_ in the non-growth season of an alpine dwarf shrubland had important effects on the interannual variation in ecosystem *A*_*max*_. In this study, the crop management regime at both sites was straw return to the field after harvest, which meant that the soil could accumulate nutrients during non-main growing season for subsequent rapid plant growth from late March because the higher T_s_ in the non-main growing season stimulated litter decomposition and faster nutrient mineralization^46^, which would improve the available nutrient supply for subsequent plant growth.

#### Why did not SWC, M-T_a_ and leaf area affect light response parameters?

The effect of SWC on photosynthetic process of ecosystem may depend on soil moisture level. Although previous studies have indicated that SWC affected light response parameters^20^, we did not find any relationships between the photosynthetic characteristics and annual mean SWC (y = −0.0089X + 0.0067, R^2^ = 0.12, P = 0.40 for *α* vs. SWC, y = 9.37X − 0.41, R^2^ = 0.43, P = 0.07 for *A*_*max*_ vs. SWC and y = −0.29X + 0.42, R^2^ = 0.03, P = 0.69 for *R*_*d*_ vs. SWC). Fu, *et al*.^1^ and Tong, *et al*.^21^ concluded that the reason that ecosystem photosynthetic characteristics hardly varied at different soil moisture levels was related to sufficient irrigation or abundant precipitation. The SWC in this study was at a moderate level because there was sufficient irrigation twice per season for winter wheat and concentrated rain between June and September. Therefore, we suggested that the suitable soil water conditions were the main reason why SWC only had a minimal effect on the light response parameters. However, a high VPD still occurred despite there being enough water, which indicated that the soil structure, soil composition, and farmland management at the two sites may have enhanced soil water holding capacity and reduced soil water evaporation.

M-T_a_ did not affect annual *α* or the other model parameters (Fig. 7c) (P > 0.05), albeit close relationship between them reported by other studies^4^. The reason that M-T_a_ affected the interannual variations in ecosystem *α*, according to previous studies, was that soil and plant enzymatic reaction rates increased exponentially with rising temperature^47^. Furthermore, the variation in LAI has also been closely correlated with T_a_^4^. However, the variations in LAI or plant enzymatic reactions were not completely affected by T_a_ in the rotations used in this study because the enzymatic reaction rates for winter wheat during the late growing stages were not affected by the T_a_ completely. T_a_ commonly reached its maximum value in August, which meant that when the winter wheat began to enter its senescence stage (mid-May to mid-June), T_a_ was beginning to continually increase. Furthermore, the correlation analysis suggested that T_a_ could only explain 9% of the change in LAI on a seasonal scale. The inconsistent decrease in the enzymatic activity of winter wheat or its LAI during the senescence stage, and the increase in T_a_ might impair the effect of M-T_a_ on the crop light response parameters on an annual scale. Li, *et al*.^2^ found a significant reduction in carbon assimilation when the T_a_ was >25 °C above cropland in the north of China. Therefore, the finding that light response parameters were not affected by T_a_ in this study might be a combined consequence of the special physiological characteristics of winter wheat and the non-moderate T_a_.

LAI is an important factor affecting plant photosynthetic capability^7^. Although ecosystem carbon uptake has been found to be positively correlated with leaf assimilation area^48^, we did not find any relationship between the annual light parameters and maximum LAI (LAI_max_) (y = −0.0015X + 0.0053, R^2^ = 0.19, P = 0.27 for *α* vs. LAI_max_, y = 0.16X + 2.98, R^2^ = 0.007, P = 0.84 for *A*_*max*_ vs. LAI_max_, y = −0.12X + 0.45, R^2^ = 0.25, P = 0.21 for *R*_*d*_ vs. LAI_max_). This result indicated that the planting density of the crops in this study might not suitable for canopy light interception because the shade effect caused by high planting densities and a large leaf area reduce ecosystem photosynthesis capacity^49^. Furthermore, plant population photosynthesis is also closely related to the duration of the plant functional leaf^50^ other than LAI.

## Conclusions

The long-term continuous eddy covariance data suggested that the among-year fluctuations in the light response parameters derived from the Michaelis-Menten rectangular hyperbola equation, i.e. ecosystem *α*, *A*_*max*_, and *R*_*d*_, were considerable during observation years at the YC and LC site. Annual mean of main – growing season VPD and *g*_*c*_ had significant effects on the annual *α* and *R*_*d*_ at the YC site but minor effects on the parameters at the LC site. The different response of the annual *α* and *R*_*d*_ to VPD and *g*_*c*_ might be related to differences in climate conditions and planting species between the two sites. A negative relationship existed between VPD and *g*_*c*_. So we indicated that the VPD inhibited *α* and *R*_*d*_ through its negative effect on *g*_*c*_. Among-year *A*_*max*_ variation was significantly affected by T_s_ of non-growing season of wheat. This study implied that sufficient rainfall and warm soil conditions during winter will enhance the ecosystem photosynthetic capacity under future climate change scenarios.

## Materials and Methods

### Site descriptions

The field experiments were conducted at Yucheng (YC) Comprehensive Experimental Station (36°57′N, 116°38′E; elevation: 23.4 m) in Shandong Province and at Luancheng (LC) Comprehensive Experimental Station (37°50′N, 114°40′E; elevation: 50.1 m) in Hebei Province. Both of the sites are within the East Asia monsoon region, which has a semi-humid and warm temperate climate. The mean annual temperature and precipitation are 13.1 °C and 528 mm, respectively, in YC (1975–2005)^35^ and 12.8 °C and 485 mm, respectively, in LC (1990–2010)^36^. Winter wheat (*Triticum aestivum* L.) and summer maize (*Zea mays* L.) were cultivated in rotation over the observational periods. At the YC site, the sowing and harvest dates for winter wheat varied from the 10^th^ October to the 29^th^ October and from the 7^th^ June to the 16^th^ June during the observation years, respectively, and the sowing and harvest dates for summer maize varied from the 13^th^ June to the 22^nd^ June and from the 30^th^ September to the 14^th^ October, respectively. At the LC site, the sowing and harvest dates for winter wheat varied from 7^th^ October to 19^th^ October and from 11^th^ June to 17^th^ June, and the sowing and harvest dates for summer maize varied from 6^th^ June to 19^th^ June and from 23^rd^ September to 2^nd^ October during the observation years, respectively. All of the straw residues from winter wheat and summer maize were returned to the field after harvest. Well water was used to irrigate the crops during the winter wheat reviving and jointing stages, and during the summer maize planting stages. Around 100–150 mm of water was added at each irrigation time^36^. The soil in the 1~20 cm layer consisted of clay loam (22.1%), silt loam (65.1%), and sandy loam (12.8%) at the YC site^51^ and was predominately sandy loam at the LC site^52^.

### Flux and meteorological measurements

The monitoring instruments and data collection methods used at the YC and LC site were similar. A three-dimensional sonic anemometer (Model CSAT 3, Campbell Scientific Inc., Logan, Utah, USA) was used to monitor fluctuations in vertical wind velocity. The CO_2_ concentration and water vapor were monitored by an open-path and fast-response infrared gas analyzer (Model LI-7500, Li-Cor Inc., Nebraka, USA). The eddy covariance instruments were placed on the towers at a height of 2.8 m and 3.5 m at the YC and LC sites, respectively. The raw flux data were collected continuously at a frequency of 10 Hz using a Campbell Scientific data logger (Model CR5000, Scientific Inc.). Average values were calculated and recorded every 30 min.

The micrometeorological measurement system consisted of a net radiometer (Model CNR-1, Kipp and Zonen, The Netherlands), a quantum sensor (LI190SB, Li-Cor Inc.), a temperature/humidity probe (Model HMP45C, Vaisala Inc., Helsinki, Finland), and an anemometer (Model AR-100, Vector Instruments), which measured net radiation, PPFD, T_a_ and relative humidity, and wind speed and direction, respectively. Other sensors measured SWC (Model CS615-L, Campbell Scientific), T_s_ (TCAV, Campbell Scientific), and rainfall (Model 52203, RM Young Inc., Traverse City, MI, USA). All the data were recorded using data loggers (Model CR23XTD, Campbell Scientific) and the data were collected every half hour^36^.

### Data processing

The half-hourly NEE (μmol CO_2_ m^−2^ s^−1^) of an ecosystem can be calculated from the covariance between the vertical wind velocity fluctuation (*w*, m s^−1^) and the CO_2_ density fluctuation (*ρ*_*c*_, μmol CO_2_ m^−3^) using the following equation:

1 $${F}_{c}=\overline{w^{\prime} {\rho ^{\prime} }_{c}}$$

where the primes denote the turbulent fluctuations (departures from the mean) and the overbar indicates the time-averaged mean (30 min). Several procedures were performed to correct the 30 min mean output data before calculating the NEE: (1) a tilt correction for the error caused by non-parallel fixation was necessary to satisfy the requirements of the eddy covariance technology^53^; (2) the eddy covariance system cannot completely capture the true turbulence when a certain number of high and low frequencies occur, which results in the loss of information compared to ideal conditions. Several situations can result in missing raw flux data, such as an inadequate sensor frequency response, separation of the instruments (particularly the sonic anemometer and the infrared gas analyzer), line averaging, and distributed sampling^54^. Therefore, aspectral correction was required to compensate for the missing raw covariance data; and (3) the Webb-Pearman-Leuning (WPL) correction was applied to correct the error caused by the transfer of heat and water vapor^55^.

After calculating the NEE, a flux data filtering process was needed to reduce uncertainties in the subsequent analyses. In this study, the apparently abnormal flux data (│NEE│ >4.0 mgCO_2_ m^−2^ s^−1^) and flux data collected under precipitation and extremely cloudy conditions were removed. After data filtering, 86% and 76% of the daytime flux data were retained for the YC and LC sites, respectively. In the NEE response to light analysis, only daytime NEE was used to describe the NEE responses to PPFD changes on an annual scale.

The ecosystem light response parameters were estimated by the Michaelis-Menten rectangular hyperbola^6^:

2 $$NEE=\frac{\alpha A{}_{\max }PPFD}{\alpha PPFD+{A}_{\max }}-{R}_{d}$$

where *α* is the initial slope of the ecosystem light-response curve, i.e. the apparent quantum yield or the apparent light-use efficiency (mgCO_2_ μmol photon^−1^), *A*_*max*_ is the maximum rate of ecosystem gross photosynthesis (GPP, mgCO_2_ m^−2^ s^−1^) at the infinite PPFD, (μmol photon m^−2^ s^−1^), and *R*_*d*_ (mgCO_2_ m^−2^ s^−1^) is the daytime ecosystem respiration. The determination coefficient (R^2^) and the confidence level (p) of the relationship between carbon flux and PPFD were also calculated.

The light response curves and the annual values for the light response parameters were fitted by the collective averages for PPFD and the incident NEE within a 100 PPFD interval using the valid daytime data (unfilled) for the main winter wheat and summer maize growing seasons. When the collective value was calculated, the value was not considered to be valid and was not fitted if the number of raw data was less than 10 per interval. In this study, due to the slight fluctuations in sowing and harvest dates for the two crops among the years, we defined the period from late March to May for winter wheat and the period from July to September for summer maize as the two main growing seasons at both sites. Other periods during the year were considered to be part of the non-main growing season and were not included when the light response curves were fitted because the carbon fluxes were close to zero from the sowing stage in October to the reviving stage during mid-March of the next year. This is due to the extremely slow growth of winter wheat. The carbon flux in June was also close to zero because it was the winter wheat harvest period and the summer maize germination stage. This study considered the winter wheat and summer maize crops as a whole ecosystem and did not analyze the differences between the two crops.

*g*_*c*_ was calculated using the Penman-Monteith equation^56^:

3 $$\frac{1}{{g}_{c}}=\frac{\rho {c}_{p}VPD}{\gamma \,LE}+[(\frac{s}{\gamma })\beta -1]\frac{1}{{g}_{a}}$$

where *ρ* (kg m^−3^) is air density, *c*_*p*_ (J kg^−1^ K^−1^) is the specific heat of the air, *s* (kPa K^−1^) is the change of saturation vapor pressure with temperature, *γ* (kPa °C^−1^) is the psychrometric constant, *β* is the Bowen ration (*H/LE*), *VPD* (kPa) is the vapor pressure deficit of air and g_*a*_ (m s^−1^) is the aerodynamic conductance of the air layer between the canopy and the flux measurement height. The g_*a*_ was calculated using:

4 $$\frac{1}{{g}_{a}}=\frac{u}{{u}^{\ast 2}}+6.2\,{u}^{\ast -0.67}$$

where *u* (m^−1^) is wind speed and u^*^ (m s^−1^) is the friction velocity^56^.

The processes used to analyze the raw flux data, fit the curves, and calculate the light response parameters were performed by MATLAB 7.4 (Mathworks Inc.).
